# Cognitive influences on biosecurity measure compliance during a global pandemic

**DOI:** 10.3389/fpsyg.2024.1306015

**Published:** 2024-05-24

**Authors:** María F. Jara-Rizzo, Nadia Soria-Miranda, Maximilian A. Friehs, Jose E. Leon-Rojas, Jose A. Rodas

**Affiliations:** ^1^Facultad de Ciencias Psicológicas, Universidad de Guayaquil, Guayaquil, Ecuador; ^2^Department of Psychology of Conflict, Risk and Safety, University of Twente, Enschede, Netherlands; ^3^School of Psychology, University College Dublin, Dublin, Ireland; ^4^Max Planck Institute for Human Cognitive and Brain Sciences, Leipzig, Germany; ^5^Escuela de Medicina, Universidad de las Américas, Quito, Ecuador; ^6^Escuela de Psicología, Universidad Espíritu Santo, Samborondón, Ecuador

**Keywords:** executive functions, cognition, compliance, biosafety measures, pandemic, COVID-19

## Abstract

**Introduction:**

During the first years of the pandemic, COVID-19 forced governments worldwide to take drastic measures to reduce the spread of the virus. Some of these measures included mandatory confinements, constant use of masks, and social distancing. Despite these measures being mandatory in many countries and the abundance of evidence on their effectiveness at slowing the spread of the virus, many people failed to comply with them.

**Methods:**

This research explored the role of cognitive factors in predicting compliance with COVID-19 safety measures across two separate studies. Building on earlier work demonstrating the relevance of cognitive processes in health behaviour, this study aimed to identify key predictors of adherence to safety guidelines during the pandemic. Utilising hierarchical regression models, we investigated the influence of age, sex, cognitive control, cognitive flexibility (Study 1), working memory, psychological health, and beliefs about COVID-19 (Study 2) on compliance to biosafety measures.

**Results:**

Demographic variables and cognitive control were significant predictors of compliance in both studies. However, cognitive flexibility and working memory did not improve the models’ predictive capacities. In Study 2, integrating measures of psychological health and beliefs regarding COVID-19 severity significantly improved the model. Further, interaction effects between age and other variables also enhanced the predictive value.

**Discussion:**

The findings emphasise the significant role cognitive control, age, psychological health, and perceptions about COVID-19 play in shaping compliance behaviour, highlighting avenues for targeted interventions to improve public health outcomes during a pandemic.

## Introduction

The global health crisis triggered by the Coronavirus disease necessitated governments worldwide to enact stringent measures aimed at curbing its spread. In the first months of the pandemic, the limited understanding of the virus’s behaviour and its elevated transmission rates lead to mandatory confinement in numerous countries. Moreover, due to the variations in SARS-CoV-2 and their potential implications for individuals with certain medical conditions, some regions persist in implementing biosecurity protocols. These include the continuous use of face masks, maintaining social distancing, and encouraging self-quarantine. However, notwithstanding these measures’ obligatory status during the pandemic, along with copious evidence substantiating their effectiveness in mitigating viral transmission (Odusanya et al., 2020; Sun and Zhai, 2020; Tso and Cowling, 2020; Ashcroft et al., 2021; Supinganto et al., 2021; Zhu and Tan, 2021), adherence remained inconsistent (Acar and Kıcali, 2022).

Numerous studies have concentrated on the elements affecting compliance with biosafety protocols such as personal hygiene, social distancing, and limited mobility or quarantines (Wismans et al., 2020; Al-Sabbagh et al., 2022). These factors range from demographic characteristics like age, gender, marital status, and number of children (Gualda et al., 2021; Uddin et al., 2021) to social elements including trust in authorities, institutions, and scientific data (Bicchieri et al., 2021; Badman et al., 2022), media engagement, political inclinations, and specific COVID-19-related conspiracy theories (Murphy et al., 2020; Painter and Qiu, 2020; Snook et al., 2021). Economic factors such as salary compensation, and support for small-scale enterprises were also explored (Bodas and Peleg, 2020). In addition, certain psychological elements have been probed, with a majority of studies examining behavioural, affective aspects and personality traits as potential predictors of compliance. For instance, habitual hygiene practices exhibited a positive correlation with social distancing (Gualda et al., 2021), whereas substance abuse displayed negative associations (Fendrich et al., 2021).

On a related note, investigations into affectivity addressed negative emotions such as functional fear and health-related anxieties (Murphy et al., 2020), in addition to anxiety surrounding self-protection and concern for at-risk demographics (Liekefett and Becker, 2021). With regard to personality traits, a study conducted during the pandemic’s apex concluded that individuals with tendencies towards kindness and conscientiousness exhibited the highest compliance with preventive guidelines; in contrast, traits of extraversion were inversely related to social distancing and travel reduction measures (Gogola et al., 2021; Krupić et al., 2021).

In the context of the coronavirus pandemic, a growing body of research has begun to explore the predictive value of cognitive processes on preventive behaviours (Xie et al., 2020; Presti et al., 2021; Znazen et al., 2021; Allan et al., 2022; Shadyab et al., 2022). For instance, Xie et al. (2020) found that working memory – a multifaceted process integral to information processing (Baddeley, 2012; Saeteros and Rodas, 2021) – significantly predicted adherence to social distancing guidelines, even when other variables such as fluid intelligence, personality, and mood were accounted for.

The examination of cognitive processes associated with the prompt adoption and adherence to biosecurity measures provides deeper insight into our capacity to adjust to novel and intricate situations, such as the ones presented during a pandemic. One pertinent construct is habituation, defined as automated behaviours that are developed through frequent repetition (Aarts et al., 1988). Habits, such as washing hands upon returning home or using the arm to cover the mouth and nose before coughing, entail numerous cognitive processes (Bargh and Gollwitzer, 1994). Cognitive flexibility (Jara-Rizzo et al., 2020) is required for learning new habits and inhibiting old ones. Moreover, emotional responses and motivation significantly impact this learning process. Other mental processes, including reasoning, short- and long-term memory, decision-making, and inhibitory control (the capacity to halt or refrain from an action), work in conjunction with cognitive flexibility to facilitate executive control (Engle et al., 1999; Miyake et al., 2000).

Biosecurity measures present new information that individuals must learn and integrate into their daily routines, which may necessitate overcoming misconceptions and automatic responses in favour of more suitable actions (Miyake et al., 2000). Here, inhibitory control is crucial to learning and fostering conceptual change (Mason and Zaccoletti, 2021). For instance, within Ecuadorian and Latin American cultures, greetings often involve close physical contact as a mark of politeness and respect. The implementation of social distancing measures necessitated a shift in these greeting habits, requiring people to greet each other from a distance and without physical contact. This significant alteration in social norms likely demanded substantial inhibitory control to suppress the instinctive impulse to greet others in traditional manners.

Several theoretical models of inhibitory control have been proposed, each with varying degrees of empirical support. For instance, Miyake et al. (2000) present a model of executive functions comprising three components: cognitive control, updating, and mental switching. In this model, cognitive control allows for the suppression of dominant responses, updating involves integrating relevant information for a task, and mental switching facilitates shifting attention between different objectives or task sets. Concurrently, Brookman-Byrne et al. (2018) highlight two forms of cognitive control: (a) behavioural inhibition, which entails motor suppression, and (b) semantic inhibition, which involves suppressing meaning in instances of conflict. Correlational studies suggest that individuals exhibiting superior cognitive control tend to perform better when tasked with scientific reasoning, and when required to overcome misleading perceptions and prior knowledge (Houdé, 2000; McNeil and Alibali, 2005). Consequently, it can be speculated that inhibitory control plays a pivotal role in facilitating the acquisition of new, functionally appropriate behavioural responses by tempering previously learned impulses.

With this in mind, we hypothesise that individuals demonstrating greater executive control (e.g., working memory, cognitive flexibility, cognitive control, and mental switching) would be more adept at adopting new behaviours mandated during the pandemic. Many of these new behaviours necessitated disrupting established habits. Therefore, across two studies we aimed to explore the roles of these cognitive processes in the adoption of novel behaviours, specifically those relating to biosafety measures, while also controlling for other variables possible implicated in the process, such as beliefs, age, sex, and mental health.

## Materials and methods

### Study 1

#### Sample

The sample comprised of 127 adults aged between 18 and 75 years (72 female; *M* = 28.37, SD = 13.35). The inclusion criteria stipulated that participants had to be at least 18 years old, possess basic computer skills, and have no psychopathology diagnosis at the time of participation. According to a power analysis, this sample size achieved a statistical power of 94% for the resulting model, taking into account its effect size and number of predictors.

#### Instruments

##### Compliance questionnaire 1

MJ-R and JR developed a questionnaire to assess compliance with biosecurity measures. This instrument, based on measures recommended by the World Health Organisation (WHO) and governments for preventing COVID-19 infections, comprises 10 items. Each item assesses the compliance level of a specific measure using a Likert scale ranging from 0 (never) to 4 (always). The items focus on behaviours such as hand hygiene, avoiding touching face, using masks, and maintaining social distance. A total compliance score is derived by summing all the responses, with higher scores indicating better compliance.

##### Colour word Stroop, computerised version

The Colour word Stroop, (MacLeod, 2005) evaluates inhibitory control by presenting a set of either congruent or incongruent stimuli. The stimuli consist of the words “rojo,” “verde,” and “azul” (red, green, and blue in Spanish, respectively) each printed in one of these three colours. Congruent stimuli feature a match between the word and the colour in which it is printed (e.g., the word “rojo” printed in red), while incongruent stimuli involve a mismatch between the word and its colour (e.g., the word “verde” printed in red). The stimuli are presented one at a time in a pseudo-random order on a black screen. Participants were asked to press a specific key based on the colour of the word, irrespective of the word itself. The task consisted of 120 trials, half of which were congruent. The dependent variable was the error rate from the incongruent trials, with higher scores indicating a decreased ability to inhibit the impulse to read the word instead of responding to the colour. This Spanish adaptation of the task has already been used in Ecuadorian population in prior studies (Rodas and Greene, 2020, 2022).

##### Probabilistic reversal learning task

This computerised task has been applied in various studies to assess decision-making, cognitive flexibility, and learning capabilities involved in processes such as the learning curve of participants (Moreno-López et al., 2015; Jara-Rizzo et al., 2020). In the task, participants are presented with a choice in each trial between two differently coloured squares—red and blue—displayed simultaneously on a black screen. Participants must select one of these squares by clicking on it. The task is organised into four distinct phases, each consisting of 40 trials. During each phase, one coloured square is arbitrarily deemed the “correct” choice. Selection of the correct square results in the award of symbolic points 80% or 70% of the time, while selecting the “wrong” square leads to the deduction of points. In the first and third phases, the red square is designated as the correct choice 80% and 70% of the time, respectively. In contrast, the blue square is the correct choice in the second and fourth phases. Participants are encouraged to accrue as many points as possible, and the overall score serves as the dependent variable.

### Study 2

#### Sample

The sample for this study consisted of 158 adults aged between 18 and 59 years (121 females; *M* = 24.79, SD = 9.88). The inclusion criteria remained the same as Study 1. According to a power analysis, this sample size achieved a statistical power of 82% for the resulting model, taking into account its effect size and number of predictors.

#### Instruments

##### Compliance questionnaire 2

This questionnaire largely mirrors the one previously described, albeit with two modifications reflecting updated circumstances. For example, in the initial questionnaire, one item enquired whether participants remained in confinement from March to June 2020. In this revised version, this question was substituted with a query about participants’ choices to remain at home with immediate family members rather than attending sizeable gatherings. Furthermore, one out of the three questions regarding face mask usage was removed.

##### Questionnaire of beliefs on the dangers of COVID-19

This questionnaire was developed by two of the authors of the current study (JR and MJ-R) to assess perceptions of the risk posed by COVID-19 to health. It is comprised of four queries: (1) perceived risk of COVID-19, (2) importance of complying with biosecurity measures, (3) extent to which the risks of COVID-19 are considered to be overstated, and (4) significance of vaccination. Participants were invited to express their level of agreement with each statement on a scale from 1 (not at all) to 5 (very much). Although the four questions within this questionnaire are related, they are analysed individually rather than yielding a cumulative score. In order to be in line with the other questions, the score from question three has been reversed, so that higher scores now represent a more positive view of biosafety measures, indicating a lower belief that the measures are overstated.

##### General Health Questionnaire 12-item version (GHQ-12)

The GFQ-12 (Vieweg and Hedlund, 1983) encompasses 12 items assessing various aspects of mental health, including sleep disturbances, low mood, and sense of self-worth. Participants respond on a Likert-type scale ranging from 0 (never) to 4 (always), culminating in a total score, where higher scores indicate greater health issues. The number of factors contained within the 12-item version is contentious, varying between one and three. In the current study, a single dimension was computed, chiefly due to the high internal consistency observed in our sample (α = 0.89). A Spanish version of the scale (Rodas and Greene, 2020) was utilised for this study, which has demonstrated good internal consistency (α = 0.8) and a satisfactory fit in factor analyses.

##### 2-Back task

The 2-back task is commonly used to evaluate working memory, especially skills relating to monitoring and updating. In this task, stimuli are presented individually and participants are required to remember the last two stimuli displayed at any given moment. This demands that participants consistently update their short-term memory as new stimuli are introduced. Participants are asked to denote with a keypress each time a stimulus matches the one presented two trials prior. The stimuli comprise letters displayed on a black screen. Each letter is visible for 500 ms with an intertrial interval of 2,500 ms. Four blocks of 25 stimuli each are presented to the participants, with each block incorporating 5 matches. Furthermore, a practice block of the same attributes is presented prior to the assessment blocks. This block provides feedback on the participants’ performance upon its completion. The dependent variable is the error rate from the four assessment blocks.

##### Colour word Stroop

The same version of the task as in Study 1 was employed.

#### Procedure for Study 1 and 2

Both studies were publicised at the Faculty of Psychology at the University of Guayaquil (Ecuador) via social media and classroom visits. Potential participants were required to contact one of the researchers, and only those who met the inclusion criteria were considered. In both studies, the assessments were conducted online using PsyToolkit (Stoet, 2010, 2017), an online platform for executing experiments and administering questionnaires. The first study took roughly 45 min to complete, and the second study approximately 60 min. Study 1 was conducted between December of 2020 and January of 2021, and Study 2 between January and February of 2022. No compensation was offered for participation in either of the studies. The sequence of the tasks and questionnaires was counterbalanced in Study 2.

All procedures performed in the current study adhered to the ethical standards of the 1964 Helsinki Declaration and its later amendments, or comparable ethical standards. The study received approval from the Scientific Committee of the University of Guayaquil, Ecuador, under the resolution “Resolución No R-CIFI-UG-SE33-159-31-07-2020.” This committee is responsible for ensuring research conducted adheres to ethical standards.

#### Analyses

In both studies, we conducted descriptive analyses, determined the internal consistency of the questionnaires by calculating Cronbach’s alpha and McDonald’s omega, evaluated the correlations between variables, and examined the predictive power of the dependent variables on the level of compliance using hierarchically constructed multiple regression models. Despite McDonald’s omega being sufficient for assessing the internal consistency of the instruments, we have also reported Cronbach’s alpha to enable comparisons with other studies as it is more commonly utilised. Pearson’s correlation coefficients were calculated among all continuous variables included in the analyses.

For the regression models, the dependent variables in Study 1 were sex and age for the initial model. Cognitive control (assessed via the Stroop task) was incorporated into a second model, and cognitive flexibility [assessed via the probabilistic reversal learning task (PRLT)] was included for the third model. The final model included an interaction between age and each of the two cognitive variables. In Study 2, the first model consisted of age and sex. Cognitive control (assessed by the Stroop task) was incorporated into a second model, working memory (measured by the 2-back task) in a third model, mental health (measured by GHQ), and beliefs (assessed by the four questions) in a fourth model. A fifth model included the interaction between age and cognitive control, age and working memory, and age and mental health. To reduce the multicollinearity within the models that incorporated interaction effects, the relevant variables were mean-centred in both studies.

## Results

Results derived from the descriptive analyses for both studies are presented in Table 1. Internal consistency was assessed for the three questionnaires employed in the two studies. In Study 1, the compliance evaluation questionnaire yielded ω = 0.776 (α = 0.762), and in Study 2, ω = 0.692 (α = 0.673). The GHQ resulted in ω = 0.894 (α = 0.893). In all instances, the instruments can be regarded as evaluating a single underlying construct, expected to be compliance in the case of the first two questionnaires and psychological health in the case of the GHQ.

**TABLE 1:** Descriptive statistics from both studies.

	Study 1		Study 2	
	**Mean**	**SD**	**Mean**	**SD**
Probabilistic reversal learning task	7.467	41.556	–	–
Stroop response time – incongruent	917.605	177.097	1,133.233	418.050
Stroop response time – congruent	807.480	165.791	991.177	342.191
Stroop – error rate	11.731	11.222	7.094	15.995
Compliance	32.669	5.240	39.722	6.177
General Health Questionnaire	–	–	14.437	6.474
2-Back task	–	–	12.025	8.481
**Beliefs on the dangers of COVID-19**				
Question 1	–	–	4.665	0.624
Question 2	–	–	4.892	0.400
Question 3	–	–	3.601	1.354
Question 4	–	–	4.646	0.749

Question 1: how dangerous is COVID-19? Question 2: how important is it to comply with biosafety measures? Question 3: to what extent are the dangers of COVID-19 exaggerated? Question 4: how important is vaccination?

Table 2 illustrates the Pearson’s correlation coefficients for the variables included in Study 1, while Table 3 outlines the correlation coefficients for Study 2.

**TABLE 2:** Correlation coefficients between variables from Study 1.

Variable	1	2	3
1. Age	–		
2. Compliance	0.040	–	
3. Cognitive control	0.159	−0.216*	–
4. Cognitive flexibility	−0.025	0.105	−0.083

*p < 0.05.

**TABLE 3:** Correlation coefficients between variables from Study 2.

Variable	1	2	3	4	5	6	7
1. Age	–						
2. Compliance	0.209**	–					
3. Mental health	−0.423***	−0.161*	–				
4. Cognitive control	−0.023	0.124	0.107	–			
5. Beliefs – question 1	0.007	0.169*	0.051	0.146	–		
6. Beliefs – question 2	0.018	0.158*	−0.009	0.047	0.364***	–	
7. Beliefs – question 3	−0.009	0.168*	0.028	−0.143	0.112	0.014	–
8. Beliefs – question 4	−0.156*	0.028	−0.006	0.029	0.248**	0.063	0.149

*p < 0.05,

**p < 0.01,

***p < 0.001.

For both studies, models were constructed using a hierarchical procedure, initiating with the demographic variables in the first model and the cognitive variables in the subsequent models. In Study 1, the initial model incorporated age and sex. This model significantly predicted compliance [*F*(2,126) = 4.78, *p* = 0.01, *R*^2^ = 0.072], with sex being the sole significant predictor. The second model introduced cognitive control and yielded a significant model [*F*(3,126) = 4.791, *p* = 0.003, *R*^2^ = 0.105] with a meaningful increase in *R*^2^ (Δ*R*^2^ = 0.033, *p* = 0.035). The third model introduced cognitive flexibility, although it did not improve its predictive capacity [*F*(4,119) = 3.528, *p* = 0.009, *R*^2^ = 0.109, Δ*R*^2^ = 0.004, *p* = 0.474]. The final model included the interaction between age and each of the cognitive variables, but this did not significantly improve the coefficient of determination [*F*(6,119) = 2.445, *p* = 0.029, *R*^2^ = 0.115, Δ*R*^2^ = 0.006, *p* = 0.699]. To alleviate multicollinearity, cognitive control and cognitive flexibility were mean-centred. Table 4 presents the results from model 2.

**TABLE 4:** Coefficients from model 2 from Study 1.

Model		*B*	SE	β	*t*	*p*
2	(Intercept)	31.685	1.287		24.627	<0.001
	Age	0.027	0.034	0.070	0.787	0.433
	Cognitive control	−0.098	0.042	−0.207	−2.297	0.023
	Sex (female)	2.315	0.951		2.435	0.016

For Study 2, the initial model incorporated age and sex and significantly predicted compliance [*F*(2,155) = 3.852, *p* = 0.023, *R*^2^ = 0.035], with age as the only significant predictor. The second model introduced inhibitory control as a predictor, yielding a significant model and an improvement in its predictive value [*F*(3,155) = 4.116, *p* = 0.008, *R*^2^ = 0.075, Δ*R*^2^ = 0.027, *p* = 0.036]. The third model introduced working memory, although it did not significantly improve its predictive value [*F*(4,154) = 3.17, *p* = 0.016, *R*^2^ = 0.078, Δ*R*^2^ = 0.001, *p* = 0.667]. The fourth model incorporated psychological health and beliefs about the dangers of COVID-19, resulting in a significant improvement in the model [*F*(9,154) = 3.118, *p* = 0.002, *R*^2^ = 0.162, Δ*R*^2^ = 0.084, *p* = 0.015]. The final model incorporated interaction effects and significantly improved the coefficient of determination [*F*(12,154) = 4.806, *p* < 0.001, *R*^2^ = 0.289, Δ*R*^2^ = 0.127, *p* < 0.001]. As in Study 1, predictors included in the interactions were mean-centred to mitigate multicollinearity. These results can be found in Table 5.

**TABLE 5:** Coefficients from model 5 from Study 2.

Model		*B*	SE	β	*t*	*p*
5	(Intercept)	27.096	6.048		4.480	<0.001
	Age	0.030	0.070	0.049	0.432	0.666
	Cognitive control	0.119	0.030	0.311	3.889	<0.001
	Working memory	−0.023	0.054	−0.033	−0.437	0.663
	Mental health	−0.148	0.077	−0.158	−1.911	0.058
	Sex (female)	1.314	1.159		1.133	0.259
**Beliefs on the dangers of COVID-19**						
	Question 1	1.105	0.800	0.114	1.380	0.170
	Question 2	1.148	1.187	0.076	0.967	0.335
	Question 3	0.769	0.333	0.171	2.307	0.022
	Question 4	−0.457	0.632	−0.056	−0.723	0.471
Age × cognitive control		0.018	0.004	0.381	4.745	<0.001
Age × working memory		−0.007	0.006	−0.087	−1.129	0.261
Age × mental health		−0.013	0.009	−0.155	−1.478	0.142

Question 1: how dangerous is COVID-19? Question 2: how important is it to comply with biosafety measures? Question 3: to what extent are the dangers of COVID-19 exaggerated? (This score is reversed.) Question 4: how important is vaccination?

## Discussion

Our two studies aimed to assess the predictive value of demographic and cognitive variables on compliance to COVID-19 safety measures, such as wearing a mask or practicing social distancing. We hypothesised that these variables could play a significant role in understanding the observed variations in individual compliance levels.

In Study 1, we found that demographic variables, specifically sex, significantly predicted compliance to safety measures. These findings align with prior literature indicating that women are often more likely to comply with preventive health behaviours compared to men (Gualano et al., 2020). However, age, another demographic variable we examined, was not a significant predictor in this study. This is somewhat surprising given that older individuals are at a higher risk from COVID-19 and therefore might be expected to comply more with safety measures.

Upon incorporating cognitive control as a predictor in our second model, we observed a significant increase in predictive power. This suggests that individual differences in cognitive control could be a key factor influencing compliance, perhaps due to its role in enabling self-regulation and the inhibition of non-compliant behaviours. However, the addition of cognitive flexibility in the third model did not result in a significant improvement in predictive power. This might imply that the ability to adapt one’s thinking in response to changing rules and recommendations is less critical for compliance than the ability to exercise cognitive control. It is also possible that the role of cognitive flexibility is more subtle. For instance, a study involving participants diagnosed with gambling disorders (Jara-Rizzo et al., 2020) found no significant differences in overall performance on the PRLT when compared to a control group of healthy adults. Nevertheless, the severity of gambling behaviour was correlated with more inefficient learning capabilities in response to altered conditions, that is, during the phases of reversed contingencies.

In Study 2, we further explored the role of cognition in adherence to safety measures. In the initial model, which accounted for age and sex as predictors of compliance, only age emerged as a significant factor. This finding is consistent with prior research indicating that older adults frequently demonstrate higher compliance with health guidelines (Barari et al., 2020). In contrast, Study 1 identified only sex as a significant predictor of compliance.

A plausible explanation for this discrepancy may lie in the evolving public perception and understanding of the disease, particularly given the 1-year gap between Study 1 and Study 2. Over time, the general population may have become more accustomed to living with COVID-19, thereby diminishing the observed differences in compliance related to sex. Moreover, increased awareness of the heightened risks faced by older individuals could have prompted them to adhere more rigorously to safety measures.

When inhibitory control was included as a predictor in the second model, the predictive power improved significantly, highlighting the potential importance of this cognitive aspect in health compliance. Interestingly, the addition of working memory as a variable in the third model did not result in a significant improvement in predictive power. This is particularly noteworthy given existing evidence suggesting that working memory processes are implicated in social distancing (Xie et al., 2020). One potential explanation for this discrepancy could be that our assessment of compliance encompasses not just social distancing but a range of behaviours, including handwashing, avoiding touching the face, and the use of face masks. It is also worth considering that the relationship between working memory and compliance with biosafety measures is complex. Working memory is a broad cognitive function related to information processing, involving a dynamic interplay among various executive, attentional, and memory processes. As such, it is not inherently linked to any specific behavioural pattern.

In the fourth model, we incorporated variables related to psychological health and beliefs about the dangers of COVID-19, resulting in a marked improvement in the model’s predictive power. Intriguingly, the only significant predictor turned out to be the belief that the implemented measures are exaggerated. Participants who did not perceive the measures as excessive were more inclined to comply, underscoring the potent influence of psychological factors and beliefs in shaping compliance behaviours.

Upon closer inspection of the belief variables, we found that neither the perceived severity of COVID-19, the relevance of vaccination, nor the importance of adhering to all measures significantly predicted compliance. Interestingly, the non-significance of beliefs regarding the importance of following biosafety measures points to a nuanced but noteworthy distinction from perceptions about exaggerated measures. This suggests that individuals may deem the level of importance as not exceedingly high but appropriately calibrated—that is, not exaggerated.

The final model incorporated interaction effects between cognition and age and saw a significant improvement in the coefficient of determination, suggesting that the impact of age on cognition might be moderating its effects on compliance. This emphasises the role of cognition over compliant behaviours since most cognitive functions tend to change with age.

The results of our studies underscore the potential importance of considering both demographic and cognitive factors when seeking to understand compliance behaviour. They suggest that interventions aimed at improving compliance may need to be tailored to specific demographic groups and may benefit from fostering cognitive skills such as cognitive and inhibitory control. However, the non-significant role of cognitive flexibility and working memory in our models also indicates that not all cognitive skills are equally relevant for compliance. Further research could explore why this might be the case.

Moreover, our findings imply the crucial role of individual beliefs about the dangers of a disease in predicting compliance behaviour. This aligns with health belief models that posit perceived threat as a critical driver of health behaviours (Rosenstock, 1974; Jones et al., 2015). As such, public health campaigns aiming to enhance compliance with preventive measures may need to focus not just on communicating factual information about health problems, but also on addressing individuals’ beliefs about the disease and its potential impact on health. Studies aimed at evaluating how individual beliefs and sociocultural aspects influence the urgent biosafety measures that must be taken in the face of health emergencies are a great precedent for planning strategies that allow the population to more easily adhere to the measures established by health organisations.

On the other hand, the non-significant role of some cognitive factors in predicting compliance might be due to the particular characteristics of the pandemic situation, where compliance with preventive measures relies not only on cognitive abilities but also on sustained motivation over time, and where the recommendations can be relatively straightforward (such as wearing a mask) and thus do not necessarily require high cognitive flexibility or working memory capacity.

It is also important to consider that the study was conducted within the specific socio-cultural context of Ecuador. Like many countries, Ecuador has its unique blend of cultural norms and health beliefs. The results highlight how demographic and cognitive factors, as well as personal beliefs about the severity and response to the COVID-19 pandemic, impact compliance with safety measures. While these findings are insightful for the Ecuadorian context, extrapolating them to other socio-cultural environments requires careful consideration. Different countries or communities might have varying degrees of emphasis on communal wellbeing, individual freedom, trust in government and health authorities, and existing health belief models, all of which could modulate the observed relationships between demographic factors, cognitive abilities, and compliance behaviours. For instance, in societies with high trust in scientific expertise and government policies, the perceived exaggeration of safety measures might have a different influence on compliance compared to contexts where scepticism towards governmental directives is prevalent. This calls for further research aimed at exploring how individual beliefs and sociocultural aspects influence urgent biosafety measures in the face of health emergencies, serving as a precedent for planning strategies that facilitate public adherence to health guidelines more effectively across diverse socio-cultural landscapes.

Limitations of our study include its reliance on self-reported compliance and the use of online assessments, which may introduce biases. Future research should aim to replicate our findings using more objective measures of compliance and in-person assessments of cognitive skills. Additionally, our study was conducted in a specific cultural and social context, and thus the generalisability of our findings to other contexts and deceases remains to be determined.

## Conclusion

In conclusion, our findings shed light on the complex interplay of demographic, cognitive, and psychological factors in predicting compliance with COVID-19 preventive measures. They underscore the importance of considering a range of factors and their interactions when designing and implementing strategies to enhance compliance. Further research is needed to deepen our understanding of these relationships and to explore their implications for public health policy and practice during the current pandemic and beyond.

## Data availability statement

The raw data supporting the conclusions of this article will be made available by the authors, without undue reservation.

## Ethics statement

The studies involving humans were approved by the Consejo de Investigación de Facultad de Ciencias Psicológicas, Universidad de Guayaquil. The studies were conducted in accordance with the local legislation and institutional requirements. The participants provided their written informed consent to participate in this study.

## Author contributions

MJ-R: Conceptualisation, Investigation, Methodology, Project administration, Writing – original draft, Writing – review & editing. NS-M: Conceptualisation, Writing – original draft. MF: Formal analysis, Writing – review & editing. JL-R: Investigation, Writing – review & editing. JR: Conceptualisation, Data curation, Formal analysis, Investigation, Methodology, Writing – original draft, Writing – review & editing.
